# Abnormality of multimodal evoked potentials in chronic inflammatory demyelinating polyradiculoneuropathy (CIDP)

**DOI:** 10.1007/s10072-020-04351-3

**Published:** 2020-03-24

**Authors:** Edyta Dziadkowiak, Maria Ejma, Malgorzata Wieczorek, Pawel Pec, Krzysztof Slotwiński, Slawomir Budrewicz, Anna Zimny, Magdalena Koszewicz

**Affiliations:** 1 Department of Neurology, Wroclaw Medical University, Wroclaw, Poland; 2 Faculty of Earth Sciences and Environmental Management, University of Wroclaw, Wroclaw, Poland; 3 Department of General Radiology, Interventional Radiology and Neuroradiology, Wroclaw Medical University, Wroclaw, Poland

**Keywords:** CIDP, Central nervous system, Evoked potentials, Neurography, Sensory conduction velocity

## Abstract

**Introduction:**

Chronic inflammatory demyelinating polyradiculoneuropathy (CIDP) is an autoimmune disease of the peripheral nervous system, sometimes including the central nervous system. The aim of the study was the assessment of the prevalence of central sensory impairment and its reliance on peripheral nerve damage in patients with CIDP.

**Material and methods:**

Multimodal (visual—VEP, brainstem auditory—BAEP, somatosensory—SEP) evoked potentials (EPs) were studied in 24 patients diagnosed with CIDP. The results were compared with neurographic parameters of sensory responses. The control group consisted of 35 healthy volunteers selected with respect to age and sex.

**Results:**

Mean latency of most components of EP were considerably prolonged in patients compared with the control group. There were no correlations between the P100 VEP latency and the peripheral sensory parameters. Statistically significant negative correlations were obtained between BAEP and SEP responses and the amplitude and sensory conduction velocity of peripheral nerves. The inter-latencies were also longer.

**Conclusions:**

The authors indicated to the possibility of central sensory involvement in patients with CIDP, especially based on the prolonged inter-latency of BAEPs with simultaneously confirmed root affection. The severity of central damage correlates with the degree of peripheral nerve impairment.

## Introduction

Chronic inflammatory demyelinating polyradiculoneuropathy (CIDP) is a rare autoimmune disorder characterized by progressive peripheral neuropathy with antibodies directed against the myelin sheath of peripheral nerves. Probable CIDP was described for the first time by Austin in 1958. It was initially characterized as a chronic inflammatory polyradiculoneuropathy by Dyck et al. in 1982 [1, 2].

Target antigens and immunological mechanisms underlying the disease have not yet been identified, while involvement of both humoral and cell-mediated immune responses has been documented [3–5].

The classic form of CIDP shows a symmetrical distribution of motor and sensory impairment, in distal and proximal parts of all four limbs, with possible involvement of cranial nerves (III, IV, VI, VII, X, XII). Dysautonomia, such as orthostatic hypotonia, dry mucous membranes, abnormal perspiration, sphincter, and sexual dysfunction, may also occur. The central nervous system is involved in about 5% of patients. Ataxia, pyramidal signs, and optic disc swelling are observed. Demyelinating brain changes can be revealed in MR in up to 20% of patients with CIDP. Previous observations support the theory of the existence of a central-peripheral inflammatory demyelinating syndrome. The combined demyelinating syndrome could be a spectrum between multiple sclerosis (MS) and CIDP or a completely separate entity [6–8].

In the typical form of CIDP, some patients report severe subjective complaints (paresthesias, pains, etc.), but in electrophysiological studies, involvement of sensory fibers is less pronounced than that of motor fibers. Miller–Fisher syndrome is a type of inflammatory neuropathy with more severe clinical and electrophysiological sensory symptoms [7, 8]. In the presented study, the authors have attempted to assess whether the central part of the sensory pathway is damaged in patients with a typical form of CIDP.

The aim of this study was the evaluation of the parameters of evoked potentials (visual, auditory, somatosensory) and their correlations with sensory conduction values of peripheral nerves in CIDP patients in order to determine the prevalence of central sensory impairment and its reliance on peripheral nerve damage.

## Material and methods

The study group consisted of 24 patients (16 male and 8 female), mean age of 60.7 years old, who fulfilled the diagnostic criteria for CIDP according to the European Federation of Neurological Societies/Peripheral Nerve Society guidelines (2010) [9]. Patients with chronic progressive CIDP were only qualified for the study. The duration of CIDP was not longer than 6 months in 13 patients, in 6—between 6 and 12 months, in 5—between 1 and 3 years.

The exclusion criteria included damage to the central nervous system of different origin, diabetes mellitus and other metabolic and systemic diseases, usage of drugs or psychostimulants, atypical forms of CIDP, multifocal motor neuropathy, and hereditary demyelinating neuropathy. Patients with visual and hearing impairment were excluded. Eleven patients had hypertension, 4—back pain in the course of spondylosis, 1—myasthenia gravis, 1—Hashimoto disease, 1—atrial fibrillation, 1—hypercholesterolemia. Two of the patients were past-smokers.

The control group consisted of 35 healthy volunteers, sex and age matched.

All patients underwent a subjective and objective neurological examination, cerebro-spinal fluid analysis, and brain CT/MRI. All patients performed spine MRI, but only 6 with contrast. We did not find any contrast enhancement of roots and nerves. In 5 of them, we tested the antibodies against ganglioside GM1, but the results were negative. We did not test the antibodies against neurofascin and contactin, because we excluded all patients with atypical CIDP.

The evoked potentials (EPs) and neurographic studies were conducted using Viking Quest equipment (Viasys Healthcare Inc., Conshohocken, PA, USA). The procedures of brainstem auditory (BAEP), somotosensory (SEP), and visual (VEP) evoked potentials were conducted according to the International Federation of Clinical Neurophysiology (IFCN) guidelines [10–12]. The study was conducted in supine position for BAEP and SEP, and sitting position for VEP, in a quiet, dimmed room at 22–24 °C. Nicolet Instrument Corporation superficial Ag/AgCl electrodes with a diameter of 10 mm were used and placed on the skin of the head according to the international 10–20 scheme. They were fixed using adhesive-conductive Ten20 Conductive paste (D.O. Weaver and Co). Each registration was performed twice in order to confirm the repeatability of the measurement.

VEPs were induced by a structural chess stimulus with alternating white and black fields emitted by a Nicolet Monitor (model NIC-1005), at a distance of 1 m. The angular size of individual squares was 1.1°, and the whole field of view was 18 × 22°. The left and right eyes were stimulated successively at a frequency of 1.88 Hz. The recording electrode was placed in the center line, on the occipital region (Oz), the reference electrode on the frontal region (Fz), and the ground electrode on the forearm. Seventy-five responses were averaged in the frequency band 1–30 Hz at the analysis time of 500 ms. The latencies of N75, P100, and N145 components, the inter-normal P100 latency difference (relative latency), and the P100-N145 amplitude were assessed. In patients with visual impairment, the examination was performed with corrective glasses.

BAEPs were obtained by stimulating (using headphones) the right and left ear with an acoustic stimulus (“click”) with a duration of 0.1 ms, a frequency of 20.3 Hz, and an intensity of 65 dB above the individually marked hearing threshold. The unexamined ear was masked with a noise of 35 dB above the hearing threshold. Responses were recorded identically using electrodes placed on the earlobe, with the reference electrode on the top of the head (A1 or A2, with respect to Cz) and the ground electrode on the forearm. A total of 2000 responses were averaged in the frequency band 150–3000 Hz in 10-ms analysis time. Absolute latencies of the I, III, and V waves, inter-latencies I–III, III–V, and I–V, and amplitudes of waves I and V were evaluated.

Prolonged inter-latencies of the I–III and/or III–V waves were considered as pathological when they were accompanied by the extension of the I–V inter-latencies. Prolonged latency of wave I was seen as abnormal when it was accompanied by changes in the latency of the subsequent auditory response. The range of mean values ± 2 SD was assumed as being correct for individual BAEP and VEP components. A difference of 50% for the P100-N145 components between the left and the right ear, and a simultaneous difference of over 50% for the I and V wave amplitudes obtained during the left and right ear stimulation were considered pathological.

SEPs were achieved by stimulating median nerves with transdermal electric impulses. Three superficial sensory electrodes were placed: one at Erb’s point (mid-clavicular point) with a contralateral reference electrode; a second at the level of the C7 vertebra with the reference electrode at the Fz central point; and the third on the head above the cortical representation of the hand, in the right (C4/P4) and left (C3/P3) parietal regions, with the contralateral reference electrode in the frontal region, at the Fz central point. The ground electrode was placed above the stimulating electrode on the forearm. The stimulating electrode on the wrist generated impulses with a duration of 100 mcs and frequency of 4.7 Hz, with the intensity resulting from thumb movement in the range of 1–2 cm. Three responses were averaged and the analysis time was 100 ms. Responses were selected and their characteristic components were interpreted. Latencies of the following SEP components were analyzed: peripheral—N9 and P10, from the brainstem—N13 and P16, cortical—N20 and P22, and interpeak latency—N20–N13, i.e., central conduction time TT. The amplitudes of N9/P10, N13/P16, and N20/P22 were also assessed. The key assumptions of the work excluded SEPs achieved by stimulating nerves in lower limbs, because of the severe lesion of peroneal and tibial nerves in most of the patients.

Standard motor conduction studies were performed in the median, ulnar nerves on the left side, in the peroneal, and tibial nerves on both sides; antidromic sensory conduction studies were performed in the left median and ulnar nerves, and both sural nerves with distal onset latency, amplitude, and conduction velocity assessment [13]. The duration of the electrical stimulation was 0.2 ms for motor and 0.1 ms for sensory fibers. Room temperature was between 21 and 23 °C, hand temperature was not less than 32 °C, and leg temperature—not less than 30 °C.

In the statistical analysis, we calculated linear correlation coefficients between four groups of parameters. We tested the normality of variables distribution using quantile-quantile plots and the Shapiro-Wilk normality test. The results indicated use of the Spearman’s rho rank correlation coefficient (13 of 26 variables do not have normal distribution) instead of the more popular Pearson correlation coefficient. *P* value ≤ 0.05 was considered significant for all tests. Statistical analysis was performed with STATISTICA *12.0* software by StatSoft.

## Results

### Analysis of clinical parameters

All patients fulfilled the criteria for probable CIDP. The average duration of clinical symptoms was 12 months: a duration of less than 6 months was in 13 patients, and over 6 months in 11 patients. Lower limb paresis dominated in 8 patients, and the paresis of 4 limbs in 10. Elevated levels of protein in the cerebrospinal fluid were found in 14 patients (mean 103.5 mg/mL). The studied subjects with CIDP had no other clinical symptoms of nervous system involvement.

We did not find any correlations between the parameters of the conduction values of the peripheral nerves, EPs, and the duration of clinical symptoms or level of protein in the cerebrospinal fluid.

### Analysis of EP parameters

We analyzed all evoked potentials separately for the left, and right side, and average values for both sides. We did not find any differences between sides.

Thirteen patients had prolonged latency of VEP, 11 of BAEP and 14 of SEP, among whom TT was prolonged (max 8.9 ms) in 6 subjects. The mean values of EP latencies significantly differed between the study group and healthy controls.

In the study group, the latencies of N75 and P100 VEP components were significantly longer than in the control group, but no differences were noted for component N145. The P100/N145 amplitude was lower, but insignificantly (Table 1). In BAEPs, the latencies of III and V waves, and the I–III, III–V, and I–V inter-latencies were also significantly longer; and the amplitude of I and V waves was significantly lower in the patients (Table 2). Mean latencies of all SEP components were considerably longer in the patients compared with the control group. Interpeak N13–N9 was significantly longer in the study group (4.87 ± 1.42 versus 3.40 ± 0.78, *p* < 0.001). Interpeak N20–N13 (central conduction time TT) did not differ significantly between groups. Mean N9/P10, N13/P16 amplitudes were lower and mean N20/P22 amplitude was higher in the patients compared with healthy individuals (Table 3).

**Table 1:** Visual evoked potentials’ parameters in CIDP patients and in the control group

VEPs		Study group *n* = 24 Mean ± SD	Control group *n* = 35 Mean ± SD	*p* value
Latency (ms)	N75	82.09 ± 13.28	70.63 ± 4.85	< 0.001
	P100	111.30 ± 10.68	100.90 ± 4.39	< 0.001
	N145	145.75 ± 18.71	141.21 ± 10.04	0.065
Amplitude (μV)	P100/N145	8.10 ± 4.06	9.41 ± 3.18	0.170

*VEPs* visual evoked potentials, *uV* microvolts, *ms* millisecond, *SD* standard deviation

**Table 2:** Brainstem evoked potentials’ parameters in CIDP patients and in the control group

BAEPs		Study group *n* = 24 Mean ± SD	Control group *n* = 35 Mean ± SD	*p* value
Latency (ms)	I	1.72 ± 0.16	1.66 ± 0.13	0.113
	III	3.98 ± 0.17	3.80 ± 0.17	< 0.001
	V	6.00 ± 0.29	5.66 ± 0.17	< 0.001
Amplitude (uV)	I	0.15 ± 0.07	0.31 ± 0.09	< 0.001
	V	0.31 ± 0.10	0.44 ± 0.12	< 0.001
Interlatency (ms)	Inter-latency I–III	2.26 ± 0.17	2.13 ± 0.10	< 0.005
	Inter-latency III–V	2.03 ± 0.25	1.86 ± 0.15	< 0.005
	Inter-latency I–V	4.28 ± 0.30	4.00 ± 0.14	<0.001

*BAEPs* brainstem evoked potentials, *uV* microvolts, *ms* millisecond, *SD* standard deviation

**Table 3:** Somatosensory evoked potentials’ parameters in CIDP patients and in the control group

SEPs		Study group *n* = 24 Mean ± SD	Control group *n* = 35 Mean ± SD	*p* value
Latency (ms)	N9	10.96 ± 1.82	9.76 ± 0.89	< 0.001
	P10	12.91 ± 2.11	11.31 ± 0.97	< 0.001
	N13	15.83 ± 2.14	13.16 ± 1.14	< 0.001
	P16	19.19 ± 2.38	16.25 ± 1.05	< 0.001
	N20	22.91 ± 2.76	19.26 ± 1.09	< 0.001
	P22	26.34 ± 3.71	22.26 ± 1.67	< 0.001
	N13–N9	4.87 ± 1.42	3.40 ± 0.78	< 0.001
	TT (N20–N13)	7.08 ± 2.17	6.10 ± 0.81	0.080
Amplitude (μV)	N9/P10	1.23 ± 0.87	2.84 ± 1.89	< 0.001
	N13/P16	0.86 ± 0.50	0.98 ± 0.37	0.351
	N20/P22	1.01 ± 0.74	0.95 ± 0.52	0.912

*SEPs* somotosensory evoked potential, *TT* transit time, *uV* microvolts, *ms* millisecond, *SD* standard deviation

### Analysis of neurographic parameters of motor and sensory responses

The results of motor conduction values are summarized in Table 4, and those of sensory conduction values of peripheral nerves in Table 5.

**Table 4:** Motor conduction values of peripheral nerves in CIDP patients

	Latency (ms)	Amplitude (mV)	Conduction velocity (m/s)
Left median nerve	5.52 ± 3.74	3.87 ± 2.57	48.17 ± 13.36
Left ulnar nerve	3.46 ± 1.25	6.24 ± 2.78	50.91 ± 12.04
Left peroneal nerve	7.13 ± 3.71	0.97 ± 1.23	27.70 ± 18.63
Left tibial nerve	7.36 ± 3.67	1.72 ± 2.33	26.26 ± 16.56
Right peroneal nerve	6.32 ± 2.95	0.79 ± 1.30	26.00 ± 17.59
Right tibial nerve	7.98 ± 6.17	1.29 ± 1.76	25.30 ± 17.49

*ms* milliseconds, *mV* millivolts, *m/s* meters per second

**Table 5:** Sensory conduction values of peripheral nerves in CIDP patients

	Latency (ms)	Amplitude (uV)	Conduction velocity (m/s)
Left median nerve	3.16 ± 0.51	14.91 ± 14.91	35.57 ± 20.46
Left ulnar nerve	2.93 ± 0.61	14.52 ± 14.80	36.87 ± 18.41
Left sural nerve	3.13 ± 0.52	3.09 ± 5.10	18.82 ± 25.73
Right sural nerve	6.34 ± 9.81	2.68 ± 4.65	15.14 ± 23.11

*ms* milliseconds, *uV* microvolts, *m/s* meters per second

### Analysis of the correlation between electroneurographic parameters and EPs

We did not find any correlations between the parameters of the motor conduction values of the peripheral nerves and EPs.

There were no correlations between the sensory conduction parameters of the peripheral nerves and VEP latencies of all components.

In BAEPs, statistically significant negative correlations were obtained between I–V and III–V inter-latencies and the amplitude and conduction velocities of peripheral sensory responses. SNAP amplitudes and sensory conduction velocities in all examined nerves negatively correlated with N20, P16, P22 SEP wave latencies, and TT.

The amplitude of sural potential also negatively correlated with mean P10 and N13 latency of SEP. SNAP amplitudes of all studied nerves and sensory conduction velocity in sural nerves positively correlated with N9–P10 SEP amplitude (Table 6).

**Table 6:** Correlation between evoked potentials and sensory nerve action potentials’ parameters

	SNAPs						
	Ulnar nerve left		Sural nerve left		Sural nerve right		
	Amplitude	Latency	Amplitude	CV	Amplitude	Latency	CV
SEPs N13	nsc	nsc	− 0.55	− 0.54	− 0.53	− 0.90	− 0.47
P16	− 0.44	nsc	− 0.64	− 0.64	− 0.63	nsc	− 0.59
N20	− 0.71	− 0.46	− 0.58	− 0.58	− 0.62	nsc	− 0.62
P22	− 0.42	nsc	nsc	nsc	nsc	nsc	nsc
TT	− 0.57	nsc	nsc	nsc	nsc	nsc	nsc
N9/P10	+ 0.68	+ 0.61	+ 0.61	+ 0.53	+ 0.64	nsc	+ 0.65
BAEPs III–V	nsc	nsc	− 0.47	nsc	− 0.54	nsc	**−** 0.46

*SEPs* somotosensory evoked potentials, *BAEPs* brainstem auditory evoked potentials, *TT* transit time, *SNAPs* sensory nerve action potentials, *CV* conduction velocity, *nsc* no significant correlation

## Discussion

CIDP is characterized by damage to peripheral nerves and nerve roots. Central nervous system involvement is rare, except Bickerstaff brainstem encephalitis. Sensory ataxic neuropathies associated with disialosyl antibodies, monoclonal proteins, and cold agglutinins were initially described by Willison et al. [14]. Ataxic neuropathies due to disialosyl antibodies can be categorized into two syndromes: CANDA (chronic ataxic neuropathy with disialosyl antibodies) and CANOMAD (chronic ataxic neuropathy, ophthalmoplegia, IgM paraproteinemia, cold agglutinin, and disialosyl antibodies). Sordo et al. [15] presented two CANOMAD patients, in whom cortical somatosensory evoked potentials by tibial nerve stimulation at the ankle were deranged, whereas responses at the popliteal fossa and sural NCS values were preserved. Sanvito et al. [16] described two cases of CANOMAD associated with optic neuropathy. They proposed the hypothesis that optic nerve damage may be related to antibody reactivity against gangliosides.

The results of a physical examination, diagnostic tests, and a standard neurographic examination enabled the diagnosis of a probable CIDP in all our patients according to the EFNS criteria, which also include the delayed response of SEPs without clinical symptoms of central nervous system damage [8, 9, 17].

We demonstrated the prolongation of latencies of different components of EPs. Similar results were obtained by Knopp et al. [17], who studied the optic pathway in CIDP. Optic pathway dysfunction was detected in half of the CIDP patients. Cabrera-Lima et al. [18] found abnormal multimodal EPs (VEPs, BAEPs, SEPs) in 8 patients out of 37 patients diagnosed with CIDP. The disturbance of EPs in CIDP patients indicated the subclinical, concomitant demyelinization of the central nervous system. MR studies performed in asymptomatic patients did not show white matter lesions. Multiple foci of high signal intensities in the cerebral white matter and around the lateral ventricles in T2-weighted MR images were infrequently seen in patients with clinical CNS manifestations, such as pyramidal or cerebellar signs. The spectrum of central and peripheral inflammatory demyelinating disease is very wide. The susceptibility for developing CIDP, MS, or both suggests a general predisposition to autoimmunity, depending on the haplotype background [19–21].

Correlations for SEP parameters obtained from the median nerve correlated with nerve conduction parameters obtained from the median and also sural nerves. These findings suggest that SEP abnormalities depend more on central than peripheral somatosensory pathway dysfunction. The prolongation of the N13–N9 inter-latency indicates root/proximal afferent impairment. Sun et al. [22] described the role of short-latency somatosensory evoked potentials (SSEP) in the diagnosis of chronic inflammatory demyelinating polyneuropathy (CIDP). SSEP examination showed conduction abnormalities in the trunk of the brachial plexus and/or the posterior roots in 7, and in the lumbosacral plexus and/or the posterior roots in 33 of 48 patients. Salhi et al. [23] used electrophysiological criteria to explore the utility of SEPs in CIDP in two groups of patients: definite or probable; and possible. There were no significant differences in SEP latencies between the groups, but the latencies of N9, N13, N7, N22, N9–N13, and N7–N22 intervals were significantly increased compared with the controls.

Another hypothesis suggests the involvement of roots within the spinal canal. The high level of sensitivity of spinal roots to immunologic disturbances may present anatomic specificities: roots of cauda equine have direct contact with cerebrospinal fluid via the spinal canal because of the lack of a brain–blood barrier. Therefore, the inflammatory process could be more pronounced [24–27].

Our results from EPs strongly correlated with sensory conduction values of peripheral nerves. We ruled out structural changes to the central nervous system on the basis of normal brain imaging. Correlations between SNAPs and EPs indicate subclinical damage to the central sensory pathways with cerebral bioelectrical changes seen in multimodal EPs. Endoneural lymphocyte and macrophage infiltrations in peripheral nerves and nerve roots in CIDP could be similar in the central nervous system and could explain central nervous system involvement.

The authors are aware of the limitations of the study. The comparison of our results with those from a second group consisting of patients with other demyelinating (e.g., hereditary) polyneuropathies would be useful for the exclusion of the influence of the peripheral nervous system on the results.

The next limitation of the study was the limited number of patients with marked variables, and the fact that the correlations were calculated for different subgroups of patients. Hence, those results suggesting statistically significant relationships must be interpreted with greater caution.

The authors indicated the possibility of central sensory involvement in patients with CIDP. The severity of central damage correlates with the degree of peripheral sensory nerve impairment. Therefore, the same pathomechanism should be taken into consideration, but it still remains unclear and needs further research.

## References

[1] Austin JH (1958). Recurrent polyneuropathies and their corticosteroid treatment; with five-year observations of a placebo-controlled case treated with corticotrophin, cortisone, and prednisone. Brain.

[2] Dyck PJ, O’Brien PC, Oviatt KF, Dinapoli RP, Daube RP, Bartleson JD, Mokri B, Swift T, Low PA, Windebank AJ (1982). Prednisone improves chronic inflammatory demyelinating polyradiculoneuropathy more than no treatment. Ann Neurol.

[3] Sanvito L, Makowska A, Mahdi-Rogers M, Hadden RDM, Peakman M, Gregson N, Nemni R, Hughes RAC (2009). Humoral and cellular immune responses to myelin protein peptides in chronic inflammatory demyelinating polyradiculoneuropathy. J Neurol Neurosurg Psychiatry.

[4] Fan C, Jin H, Hao H, Gao F, Sun Y, Lu Y, Liu Y, Lv P, Cui W, Teng Y, Huang Y (2017). Anti-ganglioside antibodies in Guillain-Barré syndrome and chronic inflammatory demyelinating polyneuropathy in Chinese patients. Muscle Nerve.

[5] PeiLim J, Devaux J, Yuki N (2014). Peripheral nerve proteins as potential autoantigens in acute and chronic inflammatory demyelinating polyneuropathies. Autoimmun Rev.

[6] Melody R, Stephen JR (2018). Chronic inflammatory demyelinating polyneuropathy: considerations for diagnosis, management, and population health. Am J Manag Care.

[7] Hu B, Zhou Y, Lu X, Xiong Q, Liu Q, Qi X, Ding W (2018). Chronic inflammatory demyelinating polyradiculoneuropathy: a case report. Medicine (Baltimore).

[8] Mendel JR, Kolkin S, Kissel JT, Weiss KL, Chakeres DW, Rammohan Kottil W (1987). Evidence for central nervous system demyelination in chronic inflammatory demyelinating polyradiculopathy. Neurol.

[9] Van den Bergh PY, Hadden RD, Bouche P, Cornblath DR, Hahn A, Illa I, Koski CL, Léger JM, Nobile-Orazio E, Pollard J, Sommer C, van Doorn PA, van Schaik IN (2010). Peripheral Nerve Society: European Federation of Neurological Societies/Peripheral Nerve Society guideline on management of chronic inflammatory demyelinating polyradiculoneuropathy: report of a joint task force of the European Federation of Neurological Societies and the Peripheral Nerve Society - first revision. Eur J Neurol.

[10] Cruccu G, Aminoff MJ, Curio G, Guerit JM, Kakini R, Mauguiere F, Rossini PM, Treede RD, Garcia-Larrea L (2008). Recommendations for the clinical use of somatosensory-evoked potentials. Clin Neurophysiol.

[11] Holder GE, Celesia GG, Miyake Y, Tobimatsu S, Weleber RG (2010). International Federation of Clinical Neurophysiology:.International Federation of Clinical Neurophysiology: recommendations for visual system testing. Clin Neurophysiol.

[12] Lee E-M, Seok HY, Park KD, Seo D-W (2018). Evoked potentials: basic requirements and guidelines for writing reports. Ann Clin Neurophysiol.

[13] Oh SJ (2003). Clinical electromyography: nerve conduction studies.

[14] Willison HJ, Paterson G, Veitch J, Inglis G, Barnett SC (1993). Peripheral neuropathy associated with monoclonal IgM anti-Pr2 cold agglutinins. J Neurol Neurosurg Psychiatry.

[15] Del Sordo E, Casali S, Ginanneschil F, Insana L, Capoccitti G, Cardinali C, Battista D, Borgheresi A, Cincotta M, Borsini W, Giannini F (2015). CANOMAD: clinical and neurophysiological findings in two cases. Clin Neurophysiol.

[16] Sanvito L, Rajabally YA (2011). Optic neuropathy associated with CANOMAD: description of 2 cases. Muscle Nerve.

[17] Knopp M, Leese RJ, Martin Lamb D, Rajabally YA (2014). Optic and auditory pathway dysfunction in demyelinating neuropathies. Acta Neurol Scand.

[18] Cabrera-Lima AV, Gutiérrez J, Martínez E, Estrada R (1999). Electrophysiological characteristics of inflammatory demyelinating chronic polyneuropathy. Rev Neurol.

[19] Ormerod IE, Waddy HM, Kermode AG, Murray NM, Thomas PK (1990). Involvement of the central nervous system in chronic inflammatory demyelinating polyneuropathy: a clinical, electrophysiological and magnetic resonance imaging study. J Neurol Neurosurg Psychiatry.

[20] Graf J, Jansen L, Ingwersen J, Ringelstein M, Harmel J, Rybak J, Kolbe R, Rhöse L, Gemerzki L, Lee JI, Klistorner A, Guthoff R, Hartung HP, Aktas O, Albrecht P (2018). Multifocal visual evoked potentials in chronic inflammatory demyelinating polyneuropathy. Ann Clin Transl Neurol.

[21] Stojkovic T, de Seze J, Hurtevent JF, Arndt C, Beaume A, Hache JC, Vermersch P (2000). Visual evoked potentials study in chronic idiopathic inflammatory demyelinating polyneuropathy. Clin Neurophysiol.

[22] Sun RD, Fu B, Jiang J (2017). Role of short-latency somatosensory evoked potentials in the diagnosis of chronić inflammatory demyelinating polyneuropathy. Zhongguo Dang Dai Er Ke Za Zhi.

[23] Salhi H, Corcia P, Remer S, Praline J (2014). Somatosensory evoked potentials in chronic inflammatory demyelinating polyradiculoneuropathy. J Clin Neurophysiol.

[24] Koutlidis RM, Ayrignac X, Pradat P-F, Le Forestier N, Leger J-M, Salachas F, Maisonobe T, Fournier E, Viala K (2014). Segmental somatosensory-evoked potentials as a diagnostic tool in chronic inflammatory demyelinating polyneuropathies, and other sensory neuropathies. Neurophysiol Clin.

[25] Hanajima R, Terao Y, Yugeta A, Hamada M, Shirota Y, Ohminami S, Nakatani-Enomoto S, Tsuji S, Ugawa Y (2010). Prominent cauda equina involvement in patients with chronic inflammatory demyelinating polyradiculoneuropathy. J Neurol Sci.

[26] Duggins AJ, McLeod JG, Pollard JD, Davies L, Yang F, Thompson EO, Soper JR (1999). Spinal root and plexus hypertrophy in chronic inflammatory demyelinating polyneuropathy. Brain.

[27] Schady W, Goulding PJ, Lecky BR, King RH, Smith CM (1996). Massive nerve root enlargement in chronic inflammatory demyelinating polyneuropathy. J Neurol Neurosurg Psychiatry.

